# Myocarditis with fulminant type 1 diabetes mellitus diagnosed by cardiovascular magnetic resonance imaging: a case report

**DOI:** 10.1186/1756-0500-6-347

**Published:** 2013-09-02

**Authors:** Katsuhiro Makino, Ikiko Nishimae, Noriyuki Suzuki, Syuya Nitta, Hiroki Saitoh, Masashi Kasao, Kazunaga Takazawa

**Affiliations:** 1 Department of Endocrinology, Tokyo Metropolitan Police Hospital, 4-22-1 Nakano, Nakano-ku, Tokyo 164-8541, Japan; 2 Department of Cardiology, Tokyo Metropolitan Police Hospital, Nakano-ku, Tokyo Japan

**Keywords:** Fulminant type 1 diabetes, Myocarditis, Diabetes, Cardiovascular magnetic resonance imaging, Diabetic ketoacidosis

## Abstract

**Background:**

Fulminant type 1 diabetes is a non-autoimmune disorder characterized by sudden onset. This complication is rarely associated with myocarditis, suggesting an involvement of viral infection. We report a patient with myocarditis who was admitted for fulminant type 1 diabetes and diagnosed using a combination of non-invasive techniques.

**Case presentation:**

We describe the case of a 25-year-old Japanese man with fulminant type 1 diabetes complicated by myocarditis. The patient was admitted with flu-like symptoms and diabetic ketoacidosis, followed by chest pain the next day. Myocardial damage was suspected based on ST-segment elevation on electrocardiogram and elevation of cardiac enzymes. However, coronary angiography revealed no abnormality in the coronary arteries. We diagnosed myocarditis by a combination of echocardiography, cardiovascular magnetic resonance imaging (CMR), as well as Thallium-201 and Iodine-123 beta-methyl iodophenyl pentadecanoic acid (Tl-201 BMIPP and I-123 BMIPP) and myocardial imaging. More importantly, CMR revealed diffuse enhancement in the subepicardium of the left ventricle with late gadolinium enhancement, consistent with myocardial edema. The patient was successfully treated, received a two-week education program on diabetes and discharged without complication.

**Conclusions:**

The rapid onset and flu-like symptoms strongly suggest the involvement of viral infection in the pathogenesis of fulminant type 1 diabetes and myocarditis. While cardiac muscle biopsy is routinely performed, this case demonstrates that a combination of non-invasive techniques, especially CMR, may successfully diagnose myocarditis in patients with fulminant type 1 diabetes.

## Background

Fulminant type 1 diabetes is a new subtype of diabetes characterized by a markedly rapid progression, an almost complete destruction of pancreatic beta cells, abnormal autoimmune response, and elevated pancreatic enzymes in the serum. While the precise mechanism of beta-cell destruction is unknown, the mononuclear cell infiltrations, detected in endocrine and exocrine pancreas, suggest an association with viral infection [1]. We report a rare case of fulminant type 1 diabetes complicated by myocarditis.

## Case presentation

A 25-year-old man consulted a local hospital for disturbed consciousness. His medical and family histories were unremarkable, except for glaucoma during childhood. He had been healthy until an episode of flu-like symptoms that occurred 5 days before admission. He lived alone, and had not been in touch with anybody for days. At the time of admission, he had hypothermia (27.8°C), elevated blood glucose (1049 mg/dl), and ketonuria. Blood gas analysis revealed an acidic pH (6.972), likely due to long-lasting metabolic abnormalities. He was diagnosed with diabetic ketoacidosis, and intravenous infusions of insulin and fluids were initiated.

The next day, the patient complained of chest pain. Since an ST-segment elevation was detected on the electrocardiograph, acute myocardial infarction was suspected. Therefore, he was transferred to our hospital. Physical findings included blood pressure, 122/64 mmHg; pulse, 95 beats/min; and temperature, 36.6°C. Pancreatic and liver enzymes were elevated. Conversely, the phosphorus level was low (1.0 mmol/L), whereas HbA1c was 6.4%, and C-peptide was undetectable in plasma (Table 1). Abdominal computed tomography revealed no abnormalities. Together, the symptoms of elevated blood glucose, near normal HbA1c, ketoacidosis, and low C-peptide level at disease onset were consistent with fulminant type 1 diabetes mellitus.

**Table 1:** Baseline blood composition

**Parameter**	**Concentration**	**Parameter**	**Concentration**
White blood cells	17,000/mm^3*^	Total protein	5.1 g/dL*
Hematocrit	37.0%*	Albumin	3.0 g/dL*
Hemoglobin	12.6 g/dL*	Aspartate aminotransferase	156 IU/L*
Platelets	180,000 /mm^3^	Alanine aminotransferase	63 IU/L*
HbA1c	6.4%	Lactate dehydrogenase	543 IU/L*
Anti-GAD antibody	5.3 U/mL*	Creatinine kinase	1753 IU/L*
Anti-IA-2 antibody	<0.4 U/mL	Creatinine kinase-MB	161 IU/L*
Fasting C-peptide	<0.03 ng/mL*	Troponin I	18.2 ng/m*L
		Urea nitrogen	24.9 mg/dL*
		Creatinine	0.76 mg/dL
		Sodium	131 mmol/L*
		Potassium	3.5 mmol/L*
		Chloride	99 mmol/L*
		Phosphorus	1.0 mmol/L*
		Amylase	216 IU/L*
		Lipase	578 IU/L*

*GAD* glutamic acid decarboxylase.

*, parameters not in the normal range.

**Figure 1:**
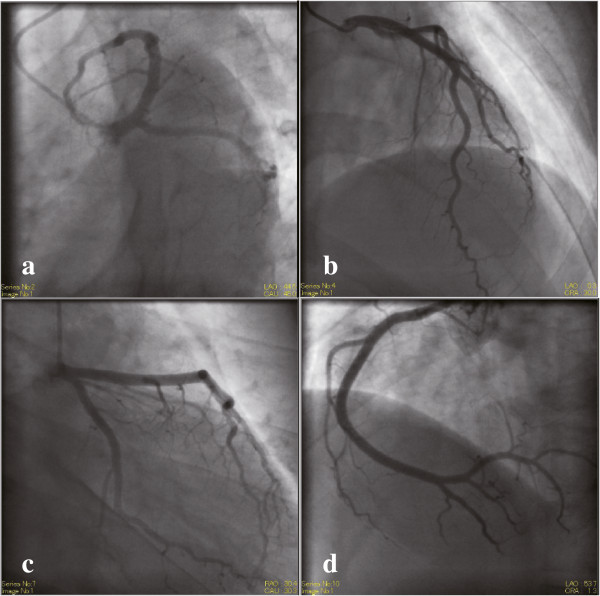
**Coronary angiography.** Absence of stenotic or obstructed lesions in the coronary arteries. **(a)** Left anterior oblique caudal (spider, LAO 45°, Caudal 45°) **(b)** Caudal (LAO 0°, Caudal 30°) **(c)** Right anterior oblique caudal (RAO 30°, Caudal 30°) **(d)** Left anterior oblique (LAO 50°, Caudal 0°).

**Figure 2:**
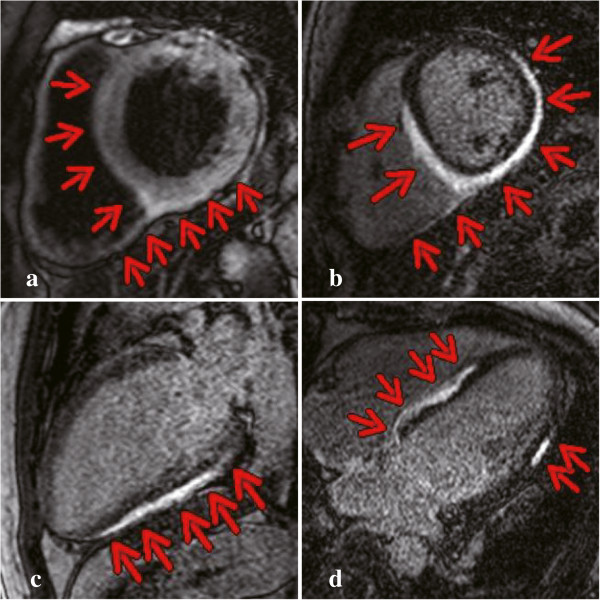
**Cardiovascular magnetic resonance imaging. (a)** T2-weighted imaging showing enhancement in the septum and posterior wall. **(b–d)** Late gadolinium enhancement showing subepicardial enhancement in the septal, anterolateral, and posterior walls.

**Figure 3:**
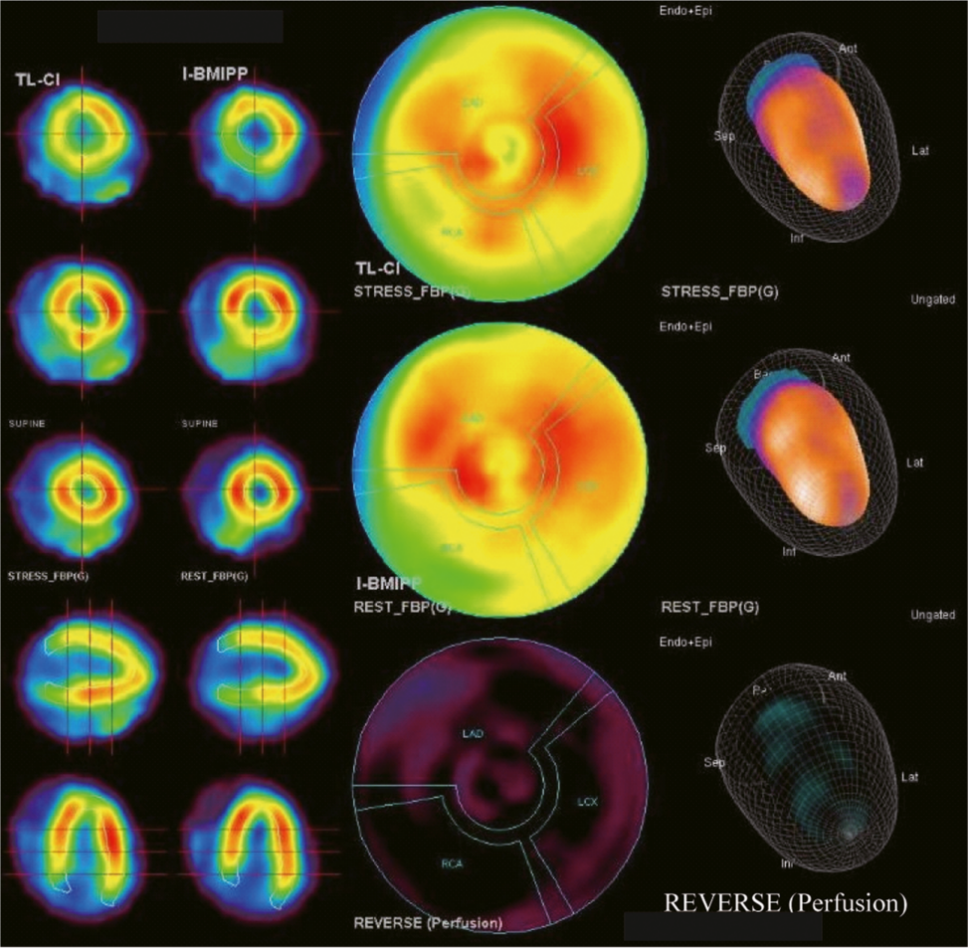
**Dual SPECT imaging using Tl-201 and I-123 beta-methyliodophenyl pentadecanoic acid (BMIPP).** Widespread distribution of tissue anomalies in the myocardium.

Electrocardiogram analysis showed ST-segment elevation in II, III, aVf, and V_4–6_. The cardiac enzymes were elevated. Echocardiography revealed diffuse hypokinesis of the left ventricle wall, and the ejection fraction was 45.3%. Coronary angiography did not detect any stenotic or obstructive lesions in the coronary arteries (Figure 1). The chest pain disappeared on the same day. On Day 3 of hospitalization, CMR revealed diffuse gadolinium myocardial enhancement in the subepicardium of the left ventricle, consistent with myocardial edema (Figure 2). Dual SPECT imaging using Tl-201 BMIPP and I-123 BMIPP showed that the defects were not restricted to a specific area in the heart (Figure 3). These findings supported the diagnosis of acute myocarditis.

The possibility of an infection was investigated using viral antibodies against influenza A and B; echovirus 3, 7, and 9; adenovirus; coxsackie A2, 9, 16, and B1-6; cytomegalovirus; and mumps. The serum antibody titer for coxsackie B4 was 64 at the time of admission, and had decreased to 32 one month later, suggesting a viral involvement in the disease.

**Figure 4:**
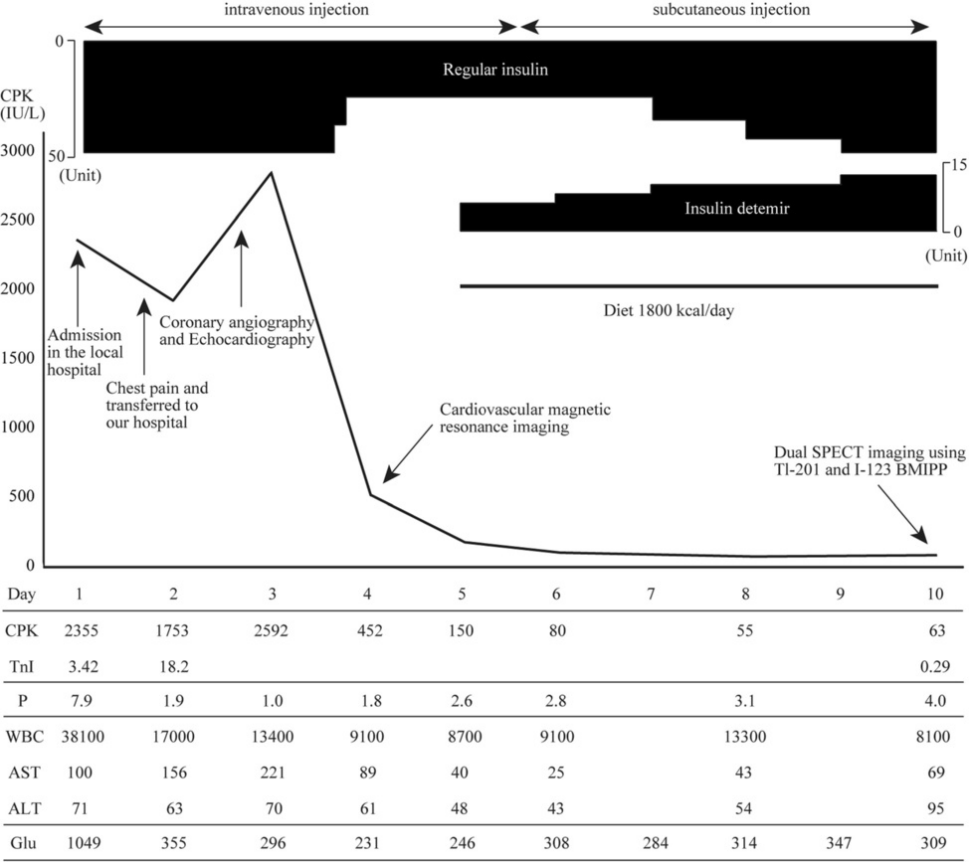
**Clinical course of imaging techniques and laboratory results.** The patient received insulin/insulin detemir treated and a low calory diet. He was monitored for serum phosphocreatine (CPK), troponin I (TnI), phosphorus (P), white blood cell counts (WBC), liver aspartate transaminase (AST), liver alanine transaminase (ALT) and glucose (Glu).

The patient was treated by carperitide and phosphorus infusions for mild heart failure and hypophosphatemia, respectively. Echocardiography performed on Day 10 showed no wall motion asynergy, and the ejection fraction recovered to 62.1%. On Day 5, intravenous insulin infusion was stopped (Figure 4), and insulin was administered subcutaneously at a scheduled time. The patient learned how to monitor blood glucose and inject insulin. After adjusting the dose of insulin, the patient received a two-week inpatient education program on diabetes mellitus. Then, he was discharged 29 days after admission.

## Discussion

Fulminant type 1 diabetes is a non-autoimmune disorder characterized by a remarkably acute onset. In most cases, the initial symptoms are flu-like symptoms, elevated pancreatic enzymes, the absence of islet-related autoantibodies, and a dramatic decrease in beta cells and alpha cells. A nationwide survey determined this variant accounts for ~ 20% of all patients with acute-onset type 1 diabetes in Japan. However, its association with myocarditis has rarely been reported. A Japanese survey revealed the involvement of viral infection, namely the coxsackie virus (types A4, 5, 6, and B1), rotavirus, cytomegalovirus, EB virus, HHV-6 and HHV-7. Cases of islet infiltrations with macrophages and T cells, as well as enterovirus RNA and Toll-like receptor-3 expression have been reported. Therefore, it has been suggested that viral infection may contribute to the development of this subtype of diabetes and trigger an anti-viral immune response [1,2]. Since our patient reported flu-like symptoms 5 days before admission, and exhibited a 2-fold decrease in coxsackie virus titer one month later, an infection-related onset of the disease is a possibility.

We considered myocarditis or Takotsubo cardiomyopathy because of the clinical symptoms of heart failure, evidence of cardiac functional perturbation, and presence of normal coronary arteries. Takotsubo cardiomyopathy, induced by stress and excess catecholamines, shows a similar clinical course as myocarditis. However, this option was ruled out because of the widespread defects detected in the myocardium by echocardiography and dual SPECT imaging [3-5]. First, Takotsubo cardiomyopathy usually affects the apical and midventricular myocardium, but does not cause diffuse hypokinesis. Second, the patchy diffuse distribution within the subepicardium on CMR is pathognomonic for myocarditis, whereas Takotsubo cardiomyopathy is generally not associated with late gadolinium enhancement [6]. Lastly, stress-induced cardiomyopathy causes milder elevations in cardiac enzyme levels than recorded in our patient [2]. On the basis of these findings, we diagnosed myocarditis. This combination of non-invasive imaging techniques reduces the need for a myocardial biopsy to diagnose myocarditis.

Mechanistically, the evidence accumulated thus far suggests that the onset of fulminant type 1 involves an immune reaction to an enterovirus. The viral infection would induce a self-perpetuating cycle of cytokine/chemokine overexpression in pancreatic beta cells, leading to apoptosis and destruction [7]. Myocarditis is also commonly induced by viral infections, including the coxsackie virus B [8]. The viruses replicate in the gut and spleen, then spreads to the heart. Their replication in the myocardium causes tissue damage amplified by an autoimmune response, leading to heart failure. Therefore, the concomitant fulminant type 1 diabetes and myocarditis exhibited by our patient may share a common etiology.

## Conclusion

Viral infections, drugs, toxins, and systemic diseases lead to myocarditis. The absence of drugs and toxins in this patient’s medical history, and the preceding flu-like symptoms, suggest that a viral infection precipitated both fulminant type 1 diabetes and myocarditis. The present case also illustrates that a combination of non-invasive techniques, including CMR, may adequately diagnose myocarditis in patients with fulminant type 1 diabetes, even in the absence of cardiac muscle biopsy.

## Consent

Written informed consent was obtained from the patient for the publication of this Case report and any accompanying images. A copy of the written consent form is available for review from the editor of this journal.

## Abbreviations

CMR: Cardiovascular magnetic resonance imaging; I-123 BMIPP: Iodine-123 beta- methyliodophenyl pentadecanoic acid; Tl-201 BMIPP: Thallium-201 beta-methyliodophenyl pentadecanoic acid.

## Competing interests

The authors declared that they have no competing interests.

## Authors’ contributions

KM, IN, NS, SN followed the patient, collected the data, reviewed the literature, collected all information, and wrote the manuscript. HS, MK and KT contributed to patient management and data collection. All authors read and approved the final version of the manuscript.
